# Editorial: Robot-Assisted Learning and Education

**DOI:** 10.3389/frobt.2020.591319

**Published:** 2020-11-09

**Authors:** Agnese Augello, Linda Daniela, Manuel Gentile, Dirk Ifenthaler, Giovanni Pilato

**Affiliations:** ^1^Institute for High Performance Computing and Networking (ICAR), National Research Council (CNR), Palermo, Italy; ^2^University of Latvia, Riga, Latvia; ^3^Institute for Educational Technology (ITD), National Research Council (CNR), Palermo, Italy; ^4^Learning, Design and Technology, University of Mannheim, Mannheim, Germany; ^5^UNESCO Deputy-Chair of Data Analytics in Higher Education Learning and Teaching, Curtin University Perth, Bentley, WA, Australia

**Keywords:** HRI (human robot interaction), technology-enhanced learning, educational robotics, social robotics, robotics for collaborative learning

Robots are increasingly being introduced in social environments to support the process of learning (e.g., Atmatzidou and Demetriadis, 2016; El Hamamsy et al., 2019; Kory-Westlund and Breazeal, 2019; Vogt et al., 2019) with different roles, such as smart teaching platforms, assistants, and in some cases also as companions and co-learners (Brown and Howard, 2014; Gordon et al., 2015; Pandey and Gelin, 2016; Belpaeme et al., 2018). Empirical research in educational robotics (ER) focuses on the adaptation of the robot behavior to specific learning needs and assessment of student learning and understanding. It is common to use robots to foster STEM and STEAM curricula (Brown and Howard, 2014; Shiomi et al., 2015; Città et al., 2017) with positive outcomes (Benitti, 2012). Research in ER have documented a greater involvement of students in learning activities, a support for critical thinking and complex problem solving as well as an increased comprehension of complex concepts and procedures, especially if the robots are endowed with a human-like appearance and social abilities (Leyzberg et al., 2012; Li, 2015). Some studies focused on the perceptions of robots and their social behavior and the consequent effects on learning (e.g., Mutlu et al., 2006; Kory and Breazeal, 2014; Michaelis and Mutlu, 2019), to support the process of understanding and memorization of concepts and the interpretation of emotional contents and social dynamics (Leite et al., 2017; Park et al., 2019; Bono et al., 2020; Conti et al., 2020).

The contributions in the Research Topic focuses on robotics approaches and architectures supporting human learning. Scaradozzi et al. apply machine learning techniques for the identification of different problem-solving pathways. Authors came to the conclusion that a “steadier incremental steps” strategy of programming correlated to a better performance in the resolution of the exercise. This supports the idea that a step by step knowledge building process is more effective than a big changes approach.

D'Amico et al. prove that introducing a robot leads to a better understanding of STEM concepts and higher participation in the activities. According to the authors, ER combines physical and mental experiences, which allow students to learn by doing, to manipulate concepts, and to embody cognition. During ER sessions, students had the opportunity to approach an idea from both an abstract and a concrete point of view. This leads to the creation of different forms of memory (semantic and procedural) and accurate episodic learning. The authors also conclude that robotics may increase motivation for learning in situations that are generally seen by children as passive and not very stimulating.

The development of cognitive strategies for the transition from exploratory actions toward intentional problem-solving in children is in the center of the development of human cognition. Charisi et al. illustrate an exploratory behavioral study to show the relationship between child-robot voluntary interaction and both the problem-solving process and performance of a child. In their study, the authors pay particular attention to the importance of exploration. Twenty children took part in the study, including 72 sessions with 113 Tower of Hanoi tasks. The platform used was a tabletop robot. The findings indicate that the children who participated in the voluntary interaction setting showed a better performance in the problem-solving activity. Implications are considered for the development of intelligent robotic systems that allow child-initiated interaction as well as targeted and not constant robot interventions.

De Haas et al. investigate how feedback from a robot can influence children's engagement and support second language learning; 72 children (5 years old) learned animal names from a humanoid in three different sessions, receiving varying types of feedback from a robot. The findings indicate that children tended to be more engaged with the robot and task when the robot used a preferred type of feedback. Implications suggest the use of robots and varying feedback in long-term interactions where engagement of children often drops.

Zhexenova et al. verify the effect of using a robot to help primary school children learning a newly-adopted script and its handwriting system. The differences between using the robot with a tablet, a tablet-only, and a teacher were not significant, revealing a similar learning effect in the three conditions. An important outcome is that children's mood improved when interacting with the robot compared to other learning aids considered in the study.

Guneysu Ozgur et al. analyze the possible role of haptic-enabled tangible robots in training visual-spatial skills. They designed an educational path to support children in learning to write cursive letters proposing tasks based on playful and collaborative activities. Starting from previous experience and applying an iterative approach, the authors adapted the activities for children with attention and visuomotor coordination issues. The experimental results gathered within occupational therapy sessions provide exciting insights (children having writing problems can improve in letter writing after the use of the system for just one session) and open up further research perspectives.

The work of Kostrubiec and Kruck belongs to the growing field of robotics for therapeutic support of children with autism syndrome. Compared to the literature, this work is characterized by the goal of collecting pieces of evidence and suggestions that can guide the realization of new robotic tools. Experimental activities have been carried out according to the ABA approach by using a spherical robotic tool not yet available on the market. The results, while showing a good acceptance by educators about the adoption of the robot, confirm some undesirable effects typical of the use of robots in these contexts, such as the difficulty of these tools to be efficient social mediators. The work highlights the need to look beyond the purely technological aspect, and to analyze in more detail how technologies integrate and interact with the adopted therapeutic approach and with the physical and social environment in which the therapies are conducted.

ER convey other important aspects. As an example, instead of focusing on the personal side of the learning process, several works investigated the use of robots in collaborative learning activities (e.g., Jung et al., 2015; Alves-Oliveira et al., 2019; Oliveira et al., 2019), to foster mutual cooperation between students strengthening the formation of social links and to support inclusive education (Catlin and Blamires, 2019; Daniela and Lytras, 2019).

Rosenberg-Kima et al. compare the effectiveness of a social-robot and a human instructor in facilitating groups in the classroom. A tablet application mediated the students' interactions to overcome the limitation of the robot in managing verbal communication. The study highlights that the physical presence of the robot and factors such as perceived intelligence, anthropomorphism, likeability, significantly influence the efficacy of the facilitator role played by the robot. Improving communication skills and providing the robot with the ability to solve situations typical of collaborative works could increase the effectiveness of these interventions.

Ponticorvo et al. show that ER can be more effective in promoting positive social ties and connections between students than other tasks when it is proposed as a group activity. A study on secondary school students (in an area strongly affected by school dropout) compares the outcomes obtained by three situations: (1) a laboratory with robots, (2) a laboratory with Scratch used for coding, and (3) a control group. The results confirm that the involvement of students in a robotics lab can effectively encourage the rise of ties among students. Furthermore, the ER, together with sociometric tools, can be used to evaluate group dynamics in a synthetic and manageable manner.

The work presented by Serholt et al. focuses the attention on troublesome situations that can occur during interaction with a robot in a classroom setting. Video analysis of children's group interactions with a robot tutee within the context of a mathematics game was conducted by examining the nature of the troubles and the strategies employed both by individual children and other involved actors to address with them. The results show as troubles mainly related to the robot's social norm violations that, although it could be traced back to technical limitations, are considered from a social interaction perspective (e.g., irrelevant comments and interruptions of the robots).

Other works shift the attention on the robot's learning side, taking inspiration from well-known learning techniques such as learning by imitation, to design algorithms enabling robots to learn procedures through observations and interaction with a human being (Tai et al., 2016; Hussein et al., 2017; Zhu and Hu, 2018).

Focusing on the recognition process of Contact States (CSs) during an assembly task Al-Yacoub et al. consider an imitation Learning approach, observing that humans can effectively manage assembly tasks by using haptic Force/Torque feedback. Collected F/T data were pre-processed and segmented. A robot learned the extracted features by temporal knowledge modeling in the symbolic domain. This makes it possible to catch complex human behaviors with models that are simpler, more compact, and with better computational performances with regards to non-symbolic models. The features are used to train a probabilistic model. Experimental trials show the effectiveness of the approach, whose main advantages are its simplicity and the minimal a priori knowledge on the geometrical characteristics on the mating parts.

## Author Contributions

AA outlined the draft by collecting the editors comments. AA, LD, MG, DI, and GP have made a substantial, intellectual contribution to the work, and approved it for publication.

## Conflict of Interest

The authors declare that the research was conducted in the absence of any commercial or financial relationships that could be construed as a potential conflict of interest.

## References

[B1] Alves-Oliveira P. Sequeira P. Melo F. S. Castellano G. Paiva A. (2019). Empathic robot for group learning: a field study. ACM Trans. Hum. Robot Interact. (Daegu) 8, 1–34. 10.1145/3300188

[B2] Atmatzidou S. Demetriadis S. (2016). Advancing students' computational thinking skills through educational robotics: a study on age and gender relevant differences. Robot. Auton. Syst. 75, 661–670. 10.1016/j.robot.2015.10.008

[B3] Belpaeme T. Kennedy J. Ramachandran A. Scassellati B. Tanaka F. (2018). Social robots for education: a review. Sci. Robot. 3:eaat5954 10.1126/scirobotics.aat5954 33141719

[B4] Benitti F. B. V. (2012). Exploring the educational potential of robotics in schools: a systematic review. Comput. Educ. 58, 978–988. 10.1016/j.compedu.2011.10.006

[B5] Bono A. Augello A. Gentile M. Gaglio S. (2020). “Social practices based characters in a robotic storytelling system,” in RO-MAN 2020 - The 29th IEEE International Symposium on Robot and Human Interactive Communication (Naples: IEEE).

[B6] Brown L. N. Howard A. M. (2014). “The positive effects of verbal encouragement in mathematics education using a social robot,” in 2014 IEEE Integrated STEM Education Conference (Princeton, NJ: IEEE), 1–5.

[B7] Catlin D. Blamires M. (2019). Designing robots for special needs education. Technol. Knowl. Learn. 24, 291–313. 10.1007/s10758-018-9378-8

[B8] Città G. Arnab S. Augello A. Gentile M. Zielonka S. I. Ifenthaler D. (2017). “Move your mind: creative dancing humanoids as support to steam activities,” in International Conference on Intelligent Interactive Multimedia Systems and Services (Vilamoura: Springer), 190–199.

[B9] Conti D. Cirasa C. Di Nuovo S. Di Nuovo A. (2020). “Robot, tell me a tale!”: a social robot as tool for teachers in kindergarten. Interact. Stud. 21, 220–242. 10.1075/is.18024.con

[B10] Daniela L. Lytras M. D. (2019). Educational robotics for inclusive education. Technol. Knowl. Learn. 24, 219–225. 10.1007/s10758-018-9397-5

[B11] El Hamamsy L. Johal W. Asselborn T. Nasir J. Dillenbourg P. (2019). “Learning by collaborative teaching: an engaging multi-party cowriter activity,” in 2019 28th IEEE International Conference on Robot and Human Interactive Communication (RO-MAN) (New Delhi: IEEE), 1–8.

[B12] Gordon G. Breazeal C. Engel S. (2015). “Can children catch curiosity from a social robot?,” in Proceedings of the Tenth Annual ACM/IEEE International Conference on Human-Robot Interaction (Portland, OR), 91–98.

[B13] Hussein A. Gaber M. M. Elyan E. Jayne C. (2017). Imitation learning: a survey of learning methods. ACM Comput. Surv. 50, 1–35. 10.1145/3054912

[B14] Jung M. F. Martelaro N. Hinds P. J. (2015). “Using robots to moderate team conflict: the case of repairing violations,” in Proceedings of the Tenth Annual ACM/IEEE International Conference on Human-Robot Interaction (Portland, OR), 229–236.

[B15] Kory J. Breazeal C. (2014). “Storytelling with robots: learning companions for preschool children's language development,” in The 23rd IEEE International Symposium on Robot and Human Interactive Communication (Edinburgh: IEEE), 643–648.

[B16] Kory-Westlund J. M. Breazeal C. (2019). A long-term study of young children's rapport, social emulation, and language learning with a peer-like robot playmate in preschool. Front. Robot. AI 6:81 10.3389/frobt.2019.00081 PMC7806079 33501096

[B17] Leite I. McCoy M. Lohani M. Ullman D. Salomons N. Stokes C. (2017). Narratives with robots: The impact of interaction context and individual differences on story recall and emotional understanding. Front. Roboti. AI 4:29 10.3389/frobt.2017.00029

[B18] Leyzberg D. Spaulding S. Toneva M. Scassellati B. (2012). “The physical presence of a robot tutor increases cognitive learning gains,” in Proceedings of the Annual Meeting of the Cognitive Science Society (Sapporo), 34.

[B19] Li J. (2015). The benefit of being physically present: a survey of experimental works comparing copresent robots, telepresent robots and virtual agents. Int. J. Hum. Comput. Stud. 77, 23–37. 10.1016/j.ijhcs.2015.01.001

[B20] Michaelis J. E. Mutlu B. (2019). “Supporting interest in science learning with a social robot,” in Proceedings of the 18th ACM International Conference on Interaction Design and Children (Boise, ID), 71–82.

[B21] Mutlu B. Forlizzi J. Hodgins J. (2006). “A storytelling robot: modeling and evaluation of human-like gaze behavior,” in 2006 6th IEEE-RAS International Conference on Humanoid Robots (Genova: IEEE), 518–523.

[B22] Oliveira R. Arriaga P. Correia F. Paiva A. (2019). “The stereotype content model applied to human-robot interactions in groups,” in 2019 14th ACM/IEEE International Conference on Human-Robot Interaction (HRI) (IEEE), 123–132.

[B23] Pandey A. K. Gelin R. (2016). “Humanoid robots in education: a short review,” in Humanoid Robotics: A Reference, eds Goswami A. Vadakkepat P., 1–16.

[B24] Park H. W. Grover I. Spaulding S. Gomez L. Breazeal C. (2019). “A model-free affective reinforcement learning approach to personalization of an autonomous social robot companion for early literacy education,” in Proceedings of the AAAI Conference on Artificial Intelligence (Honolulu, HI), Vol. 33, 687–694.

[B25] Shiomi M. Kanda T. Howley I. Hayashi K. Hagita N. (2015). Can a social robot stimulate science curiosity in classrooms? Int. J. Soc. Robot. 7, 641–652. 10.1007/s12369-015-0303-1

[B26] Tai L. Zhang J. Liu M. Boedecker J. Burgard W. (2016). A survey of deep network solutions for learning control in robotics: From reinforcement to imitation. arXiv preprint arXiv:1612.07139.

[B27] Vogt P. van den Berghe R. de Haas M. Hoffman L. Kanero J. Mamus E. (2019). “Second language tutoring using social robots: a large-scale study,” in 2019 14th ACM/IEEE International Conference on Human-Robot Interaction (HRI) (Daegu: IEEE), 497–505.

[B28] Zhu Z. Hu H. (2018). Robot learning from demonstration in robotic assembly: a survey. Robotics 7:17 10.3390/robotics7020017

